# Multitarget Antioxidant NO-Donor Organic Nitrates: A Novel Approach to Overcome Nitrates Tolerance, an Ex Vivo Study

**DOI:** 10.3390/antiox11010166

**Published:** 2022-01-16

**Authors:** Elisabetta Marini, Marta Giorgis, Marta Leporati, Barbara Rolando, Konstantin Chegaev, Loretta Lazzarato, Massimo Bertinaria, Marco Vincenti, Antonella Di Stilo

**Affiliations:** 1 Department of Drug Science and Technology, University of Turin, via Pietro Giuria 9, I-10125 Torino, Italy; marta.giorgis@unito.it (M.G.); barbara.rolando@unito.it (B.R.); Konstantin.chegaev@unito.it (K.C.); loretta.lazzarato@unito.it (L.L.); massimo.bertinaria@unito.it (M.B.); distiloantonella@gmail.com (A.D.S.); 2 Regional Antidoping & Toxicology Center “Alessandro Bertinaria”, Regione Gonzole 10/1, I-10043 Orbassano, Italy; mleporati@mauriziano.it; 3 Department of Chemistry, University of Turin, via Pietro Giuria 5, I-10125 Torino, Italy; marco.vincenti@unito.it

**Keywords:** multitarget drugs, tolerance, antioxidants, organic nitrates, aldehyde dehydrogenase 2

## Abstract

Chronic use of glyceryl trinitrate (GTN) is limited by serious side effects, such as tolerance and endothelial dysfunction of coronary and resistance arteries. Although GTN is used as a drug since more than 130 years, the mechanisms of the vasodilatory effects and of tolerance development to organic nitrates are still incompletely elucidated. New synthesized organic nitrates with and without antioxidant properties were characterized for their ex vivo tolerance profile, in order to investigate the oxidative stress hypothesis of nitrate tolerance. The organic nitrates studied showed different vasodilation and tolerance profiles, probably due to the ability or inability of the compounds to interact with the aldehyde dehydrogenase-2 enzyme (ALDH-2) involved in bioactivation. Furthermore, nitrooxy derivatives endowed with antioxidant properties did not determine the onset of tolerance, even if bioactivated by ALDH-2. The results of this study could be further evidence of the involvement of ALDH-2 in the development of nitrate tolerance. Moreover, the behavior of organic nitrates with antioxidant properties supports the hypothesis of the involvement of ROS in inactivating ALDH-2.

## 1. Introduction

Organic nitrates such as glyceryl trinitrate (GTN) are the most commonly adopted vasodilators in coronary artery diseases. The mechanism by which organic nitrates dilate blood vessels is still not fully understood. It is generally accepted that they are prodrugs that require bioactivation to yield NO or an ‘NO’-like species resulting in relaxation of the vascular smooth muscle [1,2,3]. Different enzymes have been implicated in the bioconversion of organic nitrates, in particular glutathione S-transferases, the cytochrome P450 system, xanthine oxidoreductase, and glyceraldehyde-3-phosphate dehydrogenase, although recent evidence suggests a central role for mitochondrial lipoic acid-dependent aldehyde dehydrogenase (ALDH)-2 [4,5,6]. In fact, it has been reported that the GTN bioactivation catalyzed by ALDH-2 involves the oxidation of two vicinal cysteine residues, leading to the formation of a disulfide; this mechanism implicates the requirement of a reducing agent (such as lipoic acid) for sustained catalysis [7]. Although organic nitrates are still among the most commonly prescribed medications for the treatment of angina pectoris and myocardial infarction, any hypothesis concerning the mechanism of action of these drugs must take into account the phenomenon of tolerance. In fact, the chronic efficacy of nitrates is blunted due to the development of nitrate tolerance and endothelial dysfunction [8]. It is well established that most organic nitrates cause nitrate tolerance and/or cross-tolerance to endothelium-dependent vasodilators [9]. Tolerance to nitrates is a still not well understood, complex, and multifactorial phenomenon [10], and a number of mechanisms have been proposed to explain the tolerance development [11,12]. One of the best studied and most widely accepted postulates involves the production of reactive oxygen species (ROS). The first report on a role for oxidative stress on the development of nitrate tolerance was published in 1995 by Münzel and co-workers [13]. These authors hypothesized that nitrate tolerance results from an increase in vascular superoxide, due to uncoupled endothelial nitric oxide synthase and increased activity of NADPH oxidase. Moreover, several studies showed abnormalities in the bioactivation process and in particular, in the denitration of nitrates by the ALDH-2 within the mithocondria [14,15,16]. The observation that GTN treatment triggers mitochondrial ROS production [17] leads to the proposal that ALDH-2 might be inactivated by ROS produced during sustained nitrate therapy. Indeed, ROS can oxidize ALDH-2 thiols either reversibly (disulfide form) or irreversibly (sulfonic acid); in addition, reactive oxygen species can oxidize lipoic acid causing its depletion [18,19]. In particular, a study has demonstrated that the impaired GTN biotransformation concept as well as the oxidative stress concept are closely related to each other [20]. This study showed that acute in vitro incubation of mitochondria with GTN leads to an increase in ROS production, associated with an inhibition of the mitochondrial ALDH-2. These findings were extended by in vivo observations, demonstrating that GTN treatment of rats for a 3-day period increased mitochondrial ROS production and simultaneously inhibited the activity of the enzyme. These observations supported the idea that oxidative stress may directly impair GTN biotransformation, either by oxidative inhibition of ALDH-2 or by depletion of essential repair cofactors such as lipoic acid [2,21].

**Figure 1:**
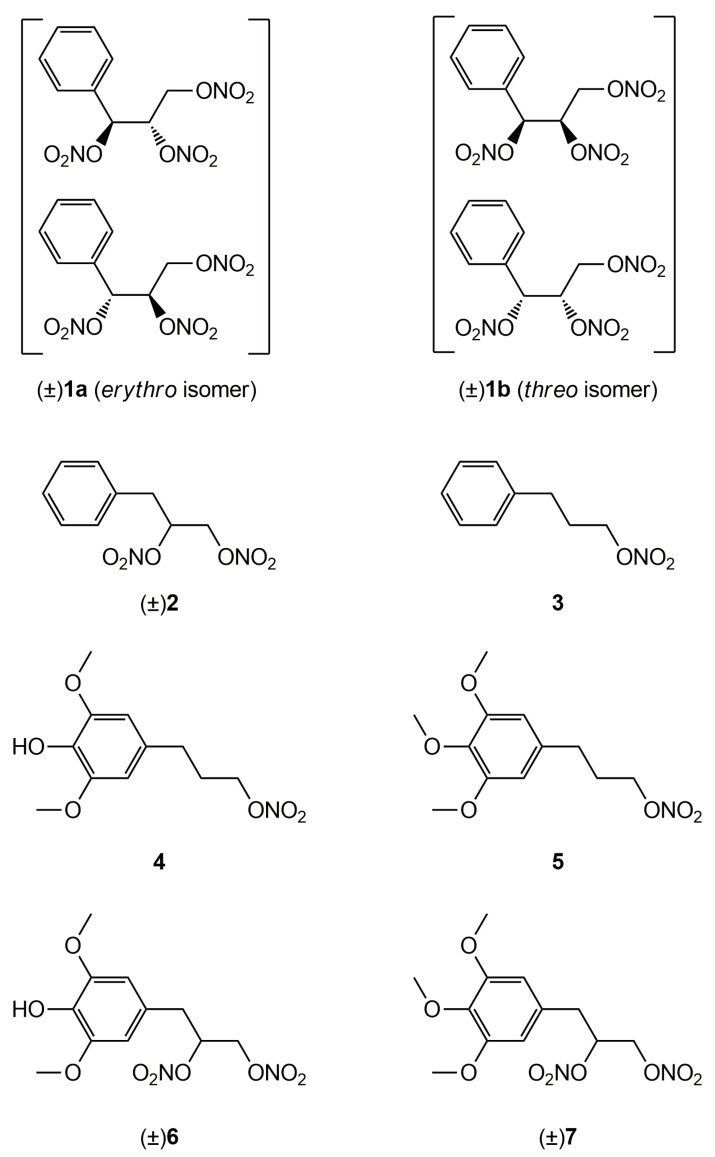
Structures of previously developed compounds **1**–**3**, **4**, **6** and newly synthesized compounds **5** and **7**.

In a previous work [22] we described the synthesis and the in vitro vasodilator profile of a new series of compounds, in which the phenyl group was introduced into the molecule of GTN; analogues obtained formally by elimination of one or two nitrooxy groups were also synthesized and characterized. On the basis of the results obtained, in this paper we report the ex vivo characterization of the tolerance profile of the nitrooxyphenylalkyl derivatives **1**-**3** (Figure 1). In the light of the oxidative stress hypothesis of nitrate tolerance, here we report also the ex vivo characterization of the tolerance profile of new organic nitrates **4** and **6** with antioxidant properties, formally obtained by joining an antioxidant phenol moiety with a nitrooxyalkyl chain. The synthesis and the antioxidant activity of these multitarget drugs were described in previous works [23,24]. The in vitro vasodilating activity of new antioxidant nitrates and their metabolic transformations are also reported here. Compounds **5** and **7** were expressly synthesized; these molecules, devoid of antioxidant activity, were taken as references, together with GTN.

## 2. Materials and Methods

### 2.1. Synthesis

Compounds **1**–**4**, **6** were synthesized as described elsewhere [22,23]. Synthesis and characterization data for the compounds **5** and **7** are reported in the Supplementary Materials.

### 2.2. Vasodilating Activity

All animals were treated humanely in accordance with recognized guidelines on experimentation; the “3 Rs” policy (99/167/EC: Council Decision of 25/1/99) of Replacement by alternative methods, Reduction of the number of animals and the Refinement of experiments were fully applied. The protocol was approved by Ministero della Salute, “Studio preliminare del profilo farmacocinetico e farmacodinamico di composti di nuova sintesi ad attività multifattoriale”. Responsible: Elisabetta Marini. Cod. n. 56105.N.ZMT, approved on 23 June 2018.

#### 2.2.1. In Vitro Experiments

Vasodilating activity was studied following a protocol published elsewhere [25], with minor modifications. Briefly, thoracic aortas were isolated from male Wistar rats weighing 180–200 g that were anaesthetized with isoflurane and killed by decapitation. The endothelium was removed and the vessels were helically cut: four to six strips were obtained from each aorta. The aortic strips were allowed to equilibrate for 120 min in organ baths containing Krebs-bicarbonate buffer with the following composition (mM): NaCl 111.2, KCl 5.0, CaCl_2_ 2.5, MgSO_4_ 1.2, KH_2_PO_4_ 1.0, NaHCO_3_ 12.0, glucose 11.1, maintained at 37 °C and gassed with 95% O_2_ 5% CO_2_ (pH 7.4), and were then contracted with 1 μM L-phenylephrine. When the response to the agonist reached a plateau, cumulative concentrations of the vasodilating agent were added. Results were expressed as EC_50_ ± SE (μM), *n* = 4–7. The effects of 1 μM benomyl [26], and 1 mM chloral hydrate (CH) [4] on relaxation were evaluated in a separate series of experiments in which the selected inhibitor was added 5 min before the contraction. With this protocol, the inhibitor was preincubated for at least 30 min before the addition of the vasodilator compound. Responses were recorded by isometric transducer (1 g resting tension) connected to the MacLab System PowerLab (ADInstruments Ltd., Oxford, UK). All synthesized compounds were dissolved in DMSO. Addition of the drug vehicle had no appreciable effect on contraction level.

#### 2.2.2. Ex Vivo Experiments

Nitrate tolerance was induced in male Wistar rats weighing 180–200 g by subcutaneous injection of 50 mg/kg/die GTN or equimolar doses of nitrooxy derivatives for 3 consecutives days. All synthesized compounds were dissolved in 200 μL of DMSO. Control animals were treated with vehicle only (200 μL), saline solution for GTN and DMSO for tested compounds. At the end of the treatment period, the animals were anaesthetized with isoflurane and killed by decapitation; thoracic aortas were removed and immediately used for functional studies, with the same protocol of in vitro experiments. Results were expressed as EC_50_ ± SE (μM), *n* = 9–21.

#### 2.2.3. Statistical Analysis

The results are presented as means ± SEM. The significance of differences was evaluated by Student’s t test for unpaired data. *p* values < 0.05 were considered significantly different. All statistical procedures were performed by commercial software (GraphPad Prism, version 7.0 from GraphPad Software Inc., San Diego, CA, USA).

### 2.3. Metabolism

#### 2.3.1. Preparation of Liver Microsomes

Wistar rats weighing 180–200 g were anaesthetized with isoflurane and were killed by decapitation; the livers were homogenized with an ice-cold 1.15% KCl solution in a Potter–Elvehjem glass–teflon homogenizer. The homogenates were centrifuged for 20 min at 8000× *g* and the supernatant fractions for 60 min at 120,000× *g*. The microsomal fractions were recovered and washed by resuspension in the KCl solution, resedimented by 60 min at 120,000× *g* and finally resuspended in 0.1 M/pH 7.4 phosphate buffer. All operations were performed at 4 °C and microsomes were stored at −80 °C. Microsomal proteins were measured by the Lowry method as modified by Schacterle [27].

#### 2.3.2. Incubation Conditions 

The derivatives **5** and **7** were incubated at 37 °C with the activated microsomal fraction for 120 min [28]. The standard incubation mixture was prepared in 0.1 M phosphate buffer, pH 7.4 with 1.3 mM MgCl_2_, 0.4 mM NADP^+^, 3.5 mM glucose 6-phosphate, 0.5 U/mL glucose 6-phosphate dehydrogenase and 100 µM of compound **5** or **7**. After pre-equilibration of the mixture at 37 °C, an appropriate volume of microsomal suspension was added to give a final protein concentration of 1 mg/mL. Control incubations were conducted without the NADPH-generating system. At fixed times (0, 60, 120 min) aliquots (200 μL) of the incubation mixture were treated with an equal volume of CH_3_CN 0.1% TFA. The precipitated proteins were separated by centrifugation, the supernatant was filtered with PTFE membrane filters 0.45 μm pore size (Alltech) and analysed by reverse phase-HPLC.

#### 2.3.3. Liver Microsomes Stability

HPLC analyses were performed with an HP 1100 chromatograph system (Agilent Technologies, Palo Alto, CA, USA) equipped with a quaternary pump (model G1311A), a membrane degasser (G1379A), and a diode-array detector (DAD) (model G1315B) integrated into the HP1100 system. Data were analyzed by the HP ChemStation system (Agilent Technologies). The analytical column was a Zorbax Eclipse XDB-C18 (150 × 4.6 mm, 5 μm particle size) (Agilent) eluted with acetonitrile/water (50/50) 0.1% TFA at a flow-rate of 1.0 mL/min. Injection volume was 20 μL (Rheodyne, Cotati, CA, USA); column effluent was monitored at 226 nm referenced against a 360 nm wavelength. The concentration of the compounds **5** and **7** and of their metabolites **4** and **6**, respectively, was calculated from the calibration curves determined in the concentration range 5–100 μM (r^2^ > 0.995).

#### 2.3.4. Metabolites Qualitative Search

The qualitative search for metabolites of compounds **5** and **7** was conducted by HPLC-MS/MS on the rat liver microsomal fraction, incubated with each compound, respectively. The separation was performed with an Agilent 1100 series liquid chromatograph (Agilent Technologies, Palo Alto, CA, USA), including a vacuum degasser, a binary pump and an autosampler. The liquid chromatograph was equipped with a Merck LiChroCART—C18 (5 μm) 150 mm × 4.6 mm column and a Phenomenex SecurityGuard 4.0 mm × 2.0 mm precolumn. The chromatographic run was carried out by a binary mobile phase of water and acetonitrile, using isocratic conditions with acetonitrile/water 0.1% formic acid (50/50) for 14 min. The flow-rate was 1 mL/min. The LC was interfaced to an Applied Biosystems API 3200 triple–quadrupole mass spectrometer (Applied Biosystems Sciex, Ontario, Canada), operating in electro spray ionization (ESI)—positive ion mode. The other MS parameters were set as follows: curtain gas: 20 psi; source gas GS1: 30 psi; source gas GS2: 30 psi; probe temperature: 350 °C; gas for collisional activation: N_2_ at 3 psi; ion spray voltage: +5000 V.

SRM analysis. The mass spectrometric signal was optimized for all investigated substances upon their synthesis as analytical standards. Setup was performed by infusion of the analyte solutions in acetonitrile at 10 μg/mL concentration. The Selected Reaction Monitoring (SRM) method was built using at least two transitions from the analytes protonated molecular ion to the corresponding fragment ions (Table 1). Then, the rat liver microsomal fraction, incubated with compound **5** or **7**, respectively, was analyzed with the same SRM method. The analyses were executed at time t = 0 and at time t = 2 h.

Product ion scan mode analysis. The search for possible metabolites was also conducted with the same chromatographic program but operating in the product ion scan mode, i.e., the protonated molecular ion of the predicted metabolites was selected with the first quadrupole (Q1), then fragmented in the intermediate cell upon collisional activation with helium molecules (Q2) and the generated product ions were analyzed by the third quadrupole (Q3) under continuous scanning conditions. 

The analyses were executed on the rat liver microsomal fraction at time t = 0 and at time t = 2 h. 

## 3. Results

### 3.1. Vasodilating Activity

#### 3.1.1. In Vitro Experiments 

**Figure 2:**
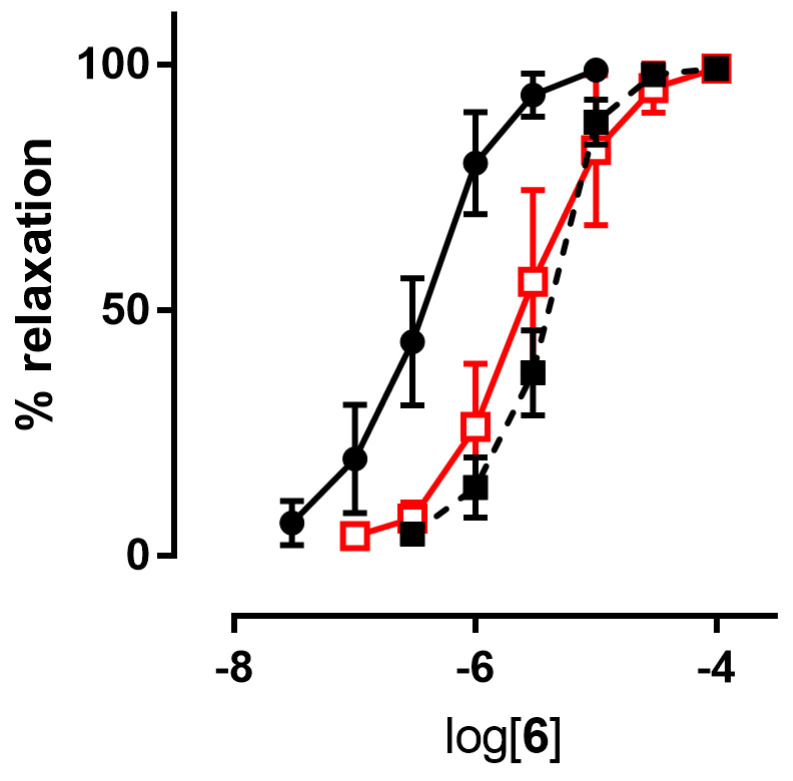
Example of **a** concentration-response curve: compound **6** in the absence (black ●) and in the presence of inhibitors of ALDH-2 (1 µM benomyl (black ■), 1 mM chloral hydrate (red □)).

Since NO predominantly modulates the tone of large conduit vessels [29,30], the vasodilator activities of the nitrooxyphenylalkyl derivatives **4**–**7**, as well as those of GTN, taken as a reference, were assessed on rat aorta strips precontracted with 1 μM L-phenylephrine. The endothelium was removed in order to study the vasodilation effects only due to the direct action of NO-donor organic nitrates. All the products were able to dilate the strips in a concentration-dependent manner. Their potencies as vasodilators, expressed as pEC_50_, are collected in Table 2. Inhibitors of ALDH-2 (chloral hydrate and benomyl) shifted the concentration–response curves of all nitrooxy derivatives to higher concentrations. An example of a concentration–response curve is reported in Figure 2. pEC_50_ values obtained in presence of inhibitors of ALDH-2 are reported in Table 2.

#### 3.1.2. Ex Vivo Experiments

**Figure 3:**
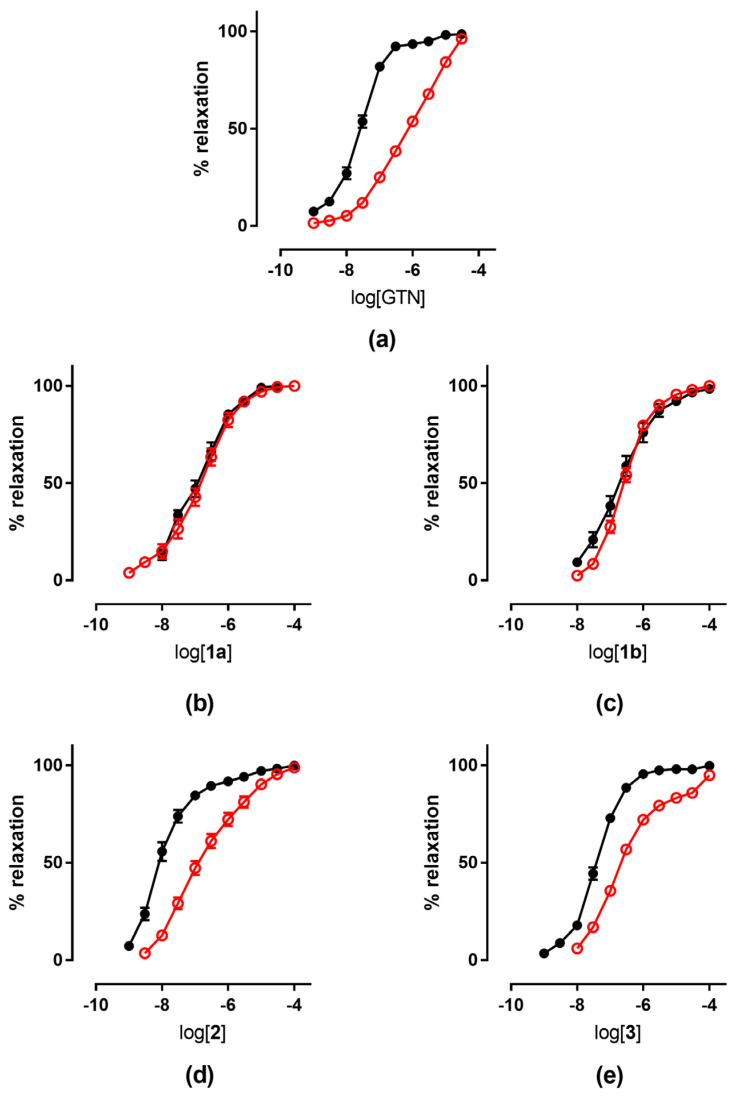
Concentration-response curves of GTN and nitrooxyphenylalkyl derivatives in control experiments (black ●) and tolerant vessels (red ○). (**a**) GTN; (**b**) compound **1a**; (**c**) compound **1b**; (**d**) compound **2**; (**e**) compound **3**.

**Figure 4:**
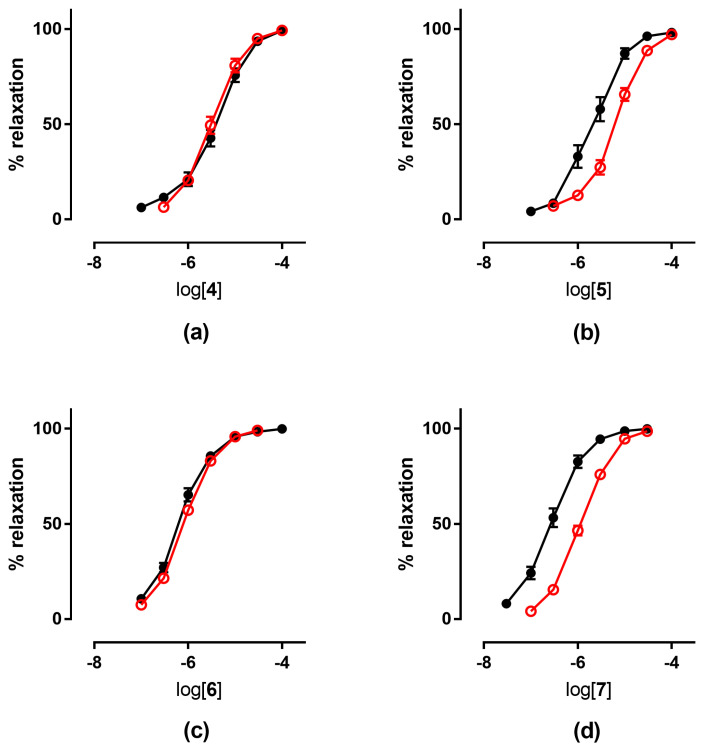
Concentration-response curves in control experiments (black ●) and tolerant vessels (red ○) of: (**a**) compound **4**; (**b**) compound **5**; (**c**) compound **6**; (**d**) compound **7**.

The tolerance profile of all the nitrates described in the present work was evaluated using a method described in the literature [31]. Nitrate tolerance was induced in rats by subcutaneous injection of 50 mg/kg/die GTN, or equimolar doses of nitrooxy derivatives, for consecutive 3 days. Control animals were treated with vehicle only, saline solution for GTN and DMSO for tested compounds. At the end of the treatment period, thoracic aortas were removed and immediately used for functional studies. pEC_50_ values are reported in Table 2. GTN and compounds **2** and **3** induced a strong development of tolerance, while vessels treated with compounds **1a** and **1b** did not show a significant rightward shift of the concentration–response curves (Figure 3). Nitrooxy derivatives **4** and **6**, endowed with antioxidant properties, did not determine the onset of tolerance (Figure 4a,c). On the contrary, the treatment with compounds **5** and **7** induced a weak development of tolerance (Figure 4b,d). 

### 3.2. Metabolism

**Figure 5:**
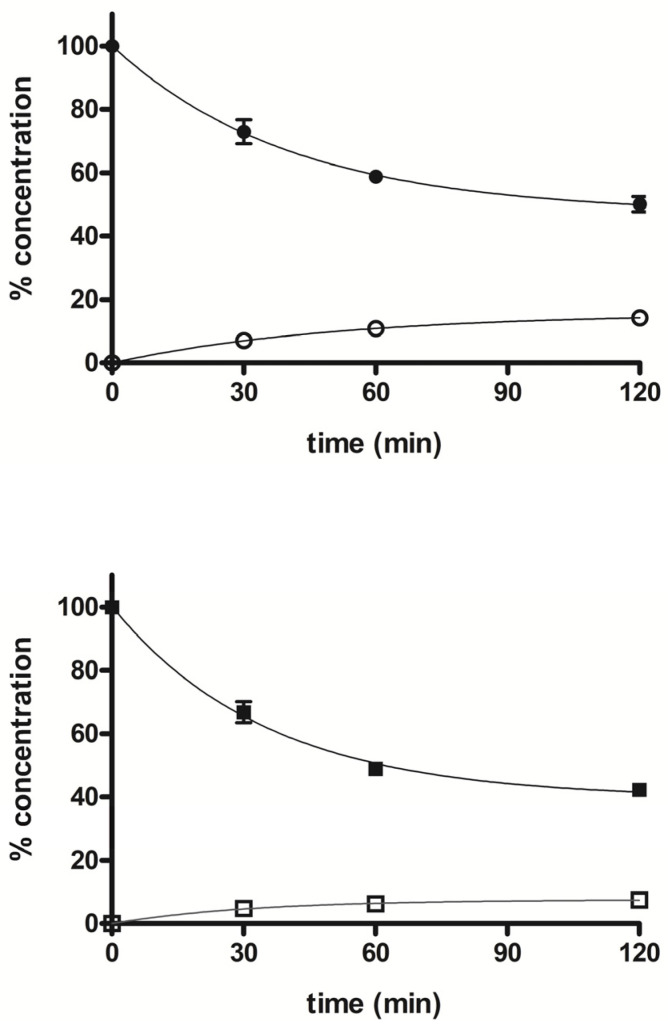
Time course of percent concentration of **5** (●), **7** (■) and its demethylated metabolites **4** (○) and **6** (□), respectively, in rat liver activated microsomal fractions during 2 h incubation; values are means ± SEM (SEM < 1; *n* = 3).

The stability profile of compounds **5** and **7** was studied in rat liver microsomal fractions in the presence of a NADPH-regenerating system. RP-HPLC analysis allowed the determination and quantification of the starting products and the expected demethylated metabolites **4** and **6,** which were formed during incubation. After 2 h incubation, the % of unchanged compounds **5** and **7** were about 50% and 42%, respectively, and the % of the demethylated derivatives **4** and **6** were about 14% and 7%, respectively. Figure 5 shows the concentration trend for all compounds (**4**–**7**) during the incubation time.

**Figure 6:**
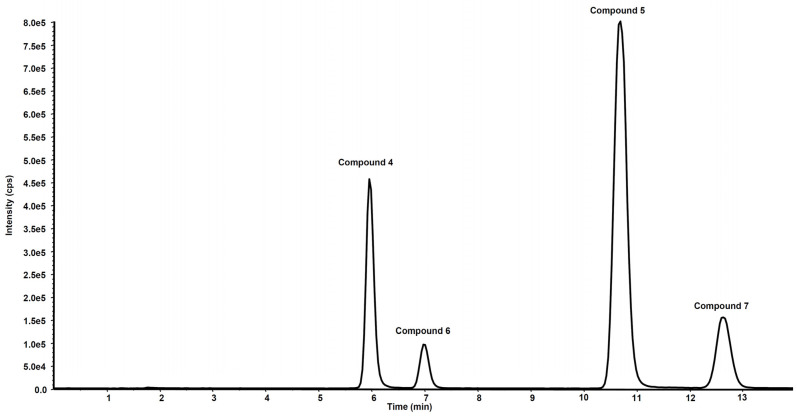
Chromatographic profile in total ion current of the ion precursor–ion products transitions reported in Table 1 of a standard solution of compounds **4**, **5**, **6** and **7** at the concentration of 10 µg/mL.

**Figure 7:**
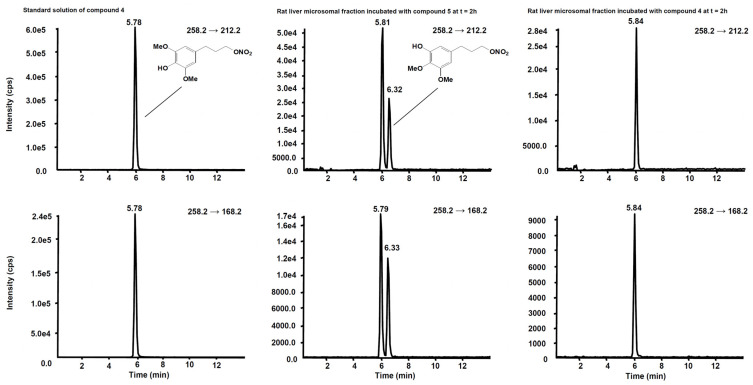
Schemes following the same formatting chromatographic profiles of the two SRM transitions distinctive for compound **4**. (**Left**) Standard solution of compound **4** at the concentration of 10 µg/mL. (**Centre**) Rat liver microsomal fraction after two hours’ incubation with compound **5**. (**Right**) Rat liver microsomal fraction after two hours’ incubation with compound **4**.

**Figure 8:**
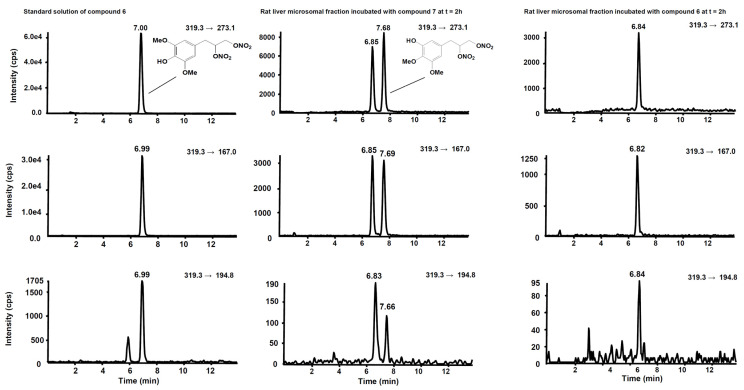
Chromatographic profiles of the three SRM transitions distinctive for compound **6**. (**Left**) Standard solution of compound **6** at the concentration of 10 µg/mL. (**Centre**) Rat liver microsomal fraction after two hours’ incubation with compound **7**. (**Right**) Rat liver microsomal fraction after two hours’ incubation with compound **6**.

Figure 6 shows the chromatogram of a standard solution of compounds **4**, **5**, **6** and **7** reported as total ion current (TIC). After two hours’ incubation of compounds **5** and **7** in the rat liver microsomal fraction, four peaks appeared in tandem mass chromatograms relative to the precursor–product ions transitions selected to detect the main metabolites, compounds **4** and **6,** respectively. Two of these signals are relative to compounds **4** and **6** while the other two peaks are possibly attributed to their structural isomers, namely compounds **4-iso** and **6-iso**, carrying the demethylated hydroxyl group in the meta-position with respect to the propyl-nitrate group. The latter peaks were not present in the chromatogram of the rat liver microsomal fraction incubated with compounds **4** or **6**, confirming that they are metabolic products of compounds **5** and **7**, respectively (Figure 7 and Figure 8).

**Figure 9:**
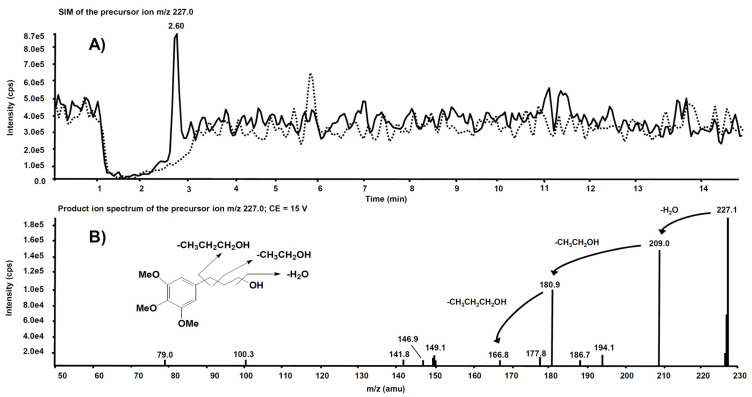
(**A**) Superimposed mass chromatograms of the *m*/*z* 227.0 precursor ion, obtained from the rat liver microsomal fraction at t = 0 (dotted line) and t = 2 h (continuous line) incubation with compound **5**. (**B**) Product ion spectrum of the selected *m*/*z* 227.0 precursor, collected at 2.60 min, from the latter analysis.

Interesting results, obtained by product ion scan mode analysis, were observed from the product ion spectra obtained after the isolation of *m*/*z* 227.0 on Q1. This precursor ion is likely to represent the molecular ion of an alleged metabolite of **5**, obtained by de-nitration of the side chain. Figure 9A reports the comparison of the *m*/*z* 227.0 chromatographic profiles obtained from the rat liver microsomal fraction before the incubation with compound **5** (dotted line) and after two hours’ incubation (continuous line). A chromatographic peak is evident at the retention time of 2.60 min only in the second profile, *viz.* after two hours’ incubation. The corresponding product ion spectrum, depicted in Figure 9B, exhibits the loss of consecutive fragments from the side chain and it is compatible with the supposed metabolite’s structure.

**Figure 10:**
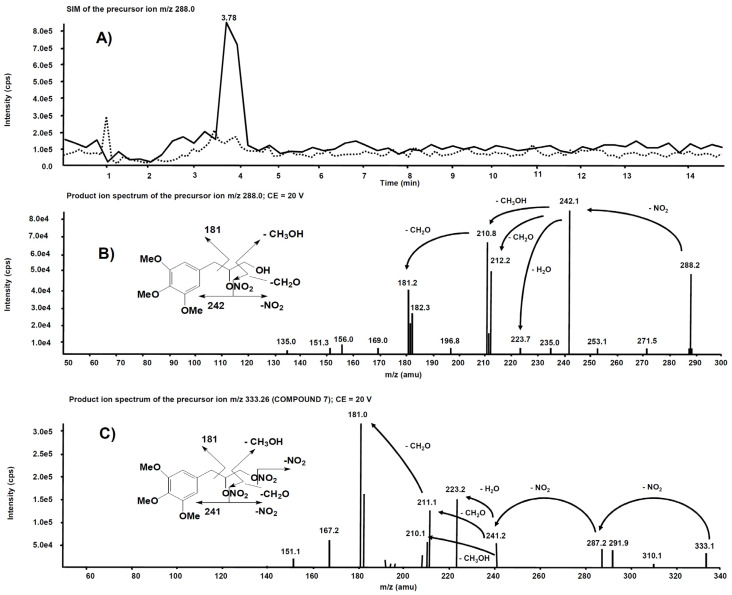
(**A**) Superimposed mass chromatograms of the *m*/*z* 288.0 precursor ion, obtained from the rat liver microsomal fraction at t = 0 (dotted line) and t = 2 h (continuous line) incubation with compound **7**. (**B**) Product ion spectrum of the selected *m*/*z* 288.0 precursor, collected at 3.78 min, from the latter analysis. (**C**) Product ion spectrum of the selected *m*/*z* 333.26, a precursor of compound **7**.

Analogue experiments were executed on the rat liver microsomal fraction incubated with compound **7**. The *m*/*z* 288.0 precursor ion isolated on Q1 corresponds to the molecular ion of the alleged metabolite **7** obtained after single de-nitration of the side chain. Figure 10A reports the comparison of the *m*/*z* 288.0 chromatographic profiles obtained from the rat liver microsomal fraction before the incubation with compound **7** and after two hours, respectively. This time, a chromatographic peak is evident at the retention time of 3.78 min only in the profile from the rat liver microsomal fraction collected after two hours’ incubation. The corresponding product ion spectrum, depicted in Figure 10B, exhibits a fragmentation similar to Figure 9B. The product ion spectrum of **7** is also reported (Figure 10C) for comparison: several fragmentations similar to those present in the product ion spectrum of its alleged metabolite are indeed observed.

The same experiment was also executed on a rat liver microsomal fraction incubated with compound **6**. The *m*/*z* 274.0 precursor ion was isolated on Q1, representing the molecular ion of a hypothetic compound **6** metabolite obtained by a single de-nitration from the side chain: in this case, no signal was observed in the chromatogram.

**Figure 11:**
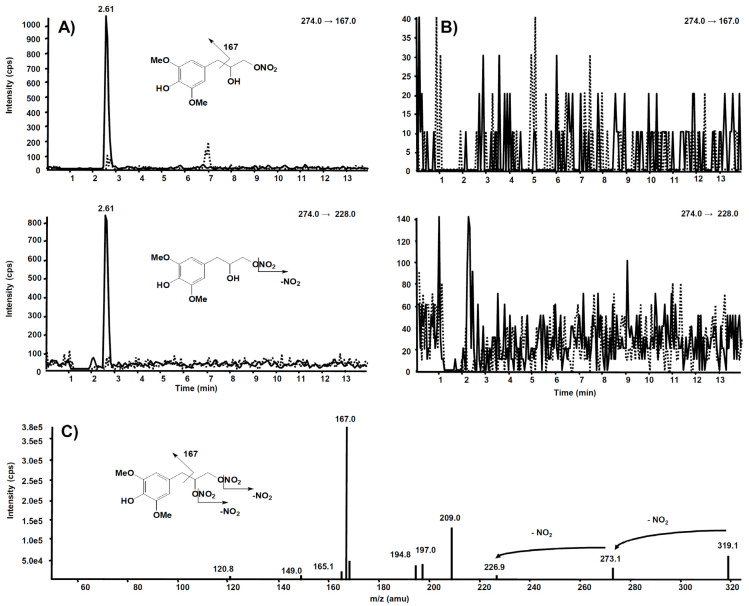
Chromatographic profiles of two plausible SRM transitions for a metabolite of compound **6**. (**A**) Rat liver microsomal fraction at t = 0 (dotted line) and t = 2 h (continuous line) incubation with compound **6**. (**B**) Rat liver microsomal fraction at t = 0 (dotted line) and t = 2 h (continuous line) incubation with compound **7**. (**C**) Product ion spectrum of the selected *m*/*z* 319.10, precursor of compound **6**.

Further and more sensitive experiments were conducted by selecting the two most probable precursor–product ion SRM transitions, namely *m*/*z* 274→228 (loss of NO_2_) and *m*/*z* 274→167 (losses from the side chain), likewise fragmentation is observed in compound **6**. Figure 11A reports the comparison between the two SRM transitions obtained from the rat liver microsomal fraction, before the incubation with compound **6** and after two hours, respectively. From this experiment of enhanced sensitivity, a high chromatographic peak is evident at the retention time of 2.61 min only in the profile collected after two hours’ incubation. The same experiment was executed on the rat liver microsomal fraction incubated with compound **7**, to confirm that *m*/*z* 274.0 corresponds to a metabolite of compound **6**, not produced from compound **7** (Figure 11B).

## 4. Discussion

Despite cardiovascular effects of GTN and organic nitrates being well established for decades, the search for novel NO-donors for clinical use is still ongoing, and the study of the mechanisms involved in tolerance development continues [32,33,34,35]. In the search for new products available both as potential drugs and for use as probes to further examine the mode of action of organic nitrates, in a previous work we characterized compounds **1**–**3** for their in vitro NO-dependent vasodilating activity [23]. Derivatives **2** and **3** showed a behavior similar to GTN, and the involvement of ALDH-2 in their bioactivation has been highlighted by experiments conducted in the presence of ALDH-2 inhibitors. Interestingly, trinitrooxy substituted derivatives **1a** and **1b** showed a completely different profile, probably because they do not interact with ALDH-2 to perform their vasodilating activity. In the light of the different vasodilation profiles, in the previous work these new nitrates were also studied in an in vitro experimental model of GTN cross-tolerance. The results of this study [22] showed that only compounds whose activity is decreased in the presence of ALDH-2 inhibitors displayed cross-tolerance with GTN, confirming the oxidative inhibition of ALDH-2 as one of the causes of this phenomenon.

On the basis of the interesting results obtained, and to learn more about the mechanisms of nitrate tolerance development, in the present work we studied the nitrooxyphenylalkyl derivatives using an ex vivo experimental model of tolerance. In this protocol, tolerance was induced in vivo, so the isolated vessel was previously made tolerant under physiological conditions, using repeated in vivo applications of GTN or nitrooxy derivatives. In this experimental model, we observed a shift to the right of the GTN biphasic concentration–response curve (Figure 3a) similar in magnitude to that previously reported in rats [31]. In order to reduce the number of animals, even if the separated enantiomers were available, racemic mixtures were used for the characterization ex vivo, since the in vitro study of the different enantiomers showed that the stereochemistry did not affect the vasodilating profile [22]. 

The results obtained in the present work for nitrooxy derivatives **1**–**3** confirmed the trend previously observed with in vitro experiments. Compounds **1a** and **1b**, respectively, erythro and threo isomers, although about 10-fold less potent than GTN, did not induce tolerance. Indeed, the concentration–response curves obtained in vessels taken from animals treated with the trinitrooxy substituted derivatives were almost identical to those obtained after the administration of DMSO alone (Figure 3b,c). Previously published in vitro studies have already shown a very low cross-tolerance between these compounds and GTN, and a profile of vasodilation very different from the reference (monophasic curve, vasodilating activity not affected by inhibitors of ALDH-2). Furthermore, the concentration–response curves herein reported from the vessels exposed in vivo to compounds **1a** and **1b** and in control experiments did not show the typical biphasic profile of GTN. 

On the contrary, compounds **2** and **3** were as potent as GTN, and they showed a similar behavior: their concentration–response curve was biphasic and tolerance development was evident (Figure 3d,e). In fact, after in vivo treatment their vasodilator potencies were reduced by about 14- and 10-fold, respectively, compared to those obtained on vessels treated with vehicle only (Table 2). The previous in vitro data for mononitrooxy and dinitrooxy substituted compounds showed a strong cross-tolerance with GTN, with a 100-fold rightward shift of the dose–response curve compared to control experiments [22]. As well, GTN in vitro showed a vasodilating response 150-fold shifted in tolerant vessels, while ex vivo (in our experiments and in the literature) the shift is less marked. These observations confirmed that the in vivo experimental models are subject to a number of variables significantly higher compared to the in vitro studies. Indeed, nitrate tolerance induced in the ex vivo model is a more complex phenomenon and it is characterized by the activation of counter-regulatory mechanisms at humoral, genomic and proteomic level, which can hardly be reproduced in vitro. Finally, all nitrooxyphenylalkyl derivatives presented an ex vivo vasodilating profile similar to previously published in vitro experiments; the results thus obtained showed that a nitrate, when bioactivated by ALDH-2, can induce tolerance.

In recent years, several studies have hypothesized that nitrate tolerance might be due to the oxidative inactivation of ALDH-2 [19,21,36]. Moreover, dysregulation of key enzymes and the resulting production of superoxide anion (O_2_^●−^), which rapidly traps NO to generate peroxynitrite (-OONO), appear to be also crucial for the endothelial dysfunction caused by chronic nitrate treatment [37,38]. Antioxidants preserved the sensitivity of the vasculature to organic nitrates in different animal models, but proved ineffective in clinical settings [39,40]. However, promising results emerged from the study of mitochondriotropic quercetin derivatives [41,42]. These compounds reduced in vitro GTN tolerance development and restored the pharmacological response to acetylcholine. Indeed, mitochondrial accumulation of these derivatives reduced the concentration of radicals responsible for the development of tolerance and endothelial dysfunction caused by chronic nitrate treatment. In another work, the administration of the selective peroxynitrite decomposition catalyst MnTBAP (10 mg/kg, i.p.) significantly restored the hypotensive effect of bolus injection of GTN in rats made tolerant to organic nitrates via chronic administration of isosorbide-5-mononitrate (IS-5-MN). These findings confirmed the role of peroxynitrite overproduction in the development of tolerance to vascular responses induced by organic nitrates [43].

On this basis, in order to deepen the oxidative stress hypothesis of nitrate tolerance, in the present work we decided to use a completely different and innovative approach: molecules structurally similar to nitrooxyphenyl derivatives **2** and **3**, but endowed with antioxidant properties, were studied in the ex vivo experimental model. In a previous work, compounds **4** and **6** were tested on rat liver microsomes as inhibitors of lipid peroxidation induced by iron/ascorbate; both the NO-donor phenols inhibited the production of TBARS in a concentration-dependent manner with IC_50_ values lower than that of the reference phenol, 5.9 (5.5–6.4) µM and 5.4 (5.1–5.8) versus 18 (17–20) µM, respectively [23]. In another work, the scavenger properties of **4** and **6** were studied, and the kinetic parameter log*Z* (measured from DPPH absorbance quenching in the first 15 s of reaction) was reported [24]. Log*Z* values obtained for **4**, **6** and the reference phenol were similar: 3.11, 2.78 and 3.24, respectively. Therefore, derivatives **4** and **6** have ideal characteristics for the study of the involvement of ROS in inactivating ALDH-2: both possess a good antioxidant activity and are potentially able to induce tolerance, such as the dinitrooxy and mononitrooxyphenyl derivatives **2** and **3**. Compounds **5** and **7**, devoid of antioxidant activity, were taken as references.

Compounds **4**–**7** were first studied in vitro to characterize their NO-dependent vasodilating activity. The introduction of hydroxyl and methoxyl substituents into the nitrooxyphenylalkyl derivatives did not modify the in vitro vasodilating profile previously observed for compounds **2** and **3**. All new compounds, although less potent than derivatives **2** and **3**, displayed a behavior reminiscent of GTN in the presence of the ALDH-2 inhibitors used in our experiments. Indeed, in tissues preincubated with benomyl and chloral hydrate the concentration–response curve was shifted rightwards about 4- to 10-fold (Table 2, Figure 2). These results showed the involvement of ALDH-2 in their bioactivation.

Ex vivo experiments highlighted that nitrooxy derivatives **4** and **6** did not determine the onset of tolerance (Figure 4a,c). Under the same experimental conditions, mononitrooxy and dinitrooxyphenyl derivatives **2** and **3** induced a strong development of tolerance (Figure 3d,e). Therefore, the presence in **4** and **6** of an antioxidant function able to scavenge intracellular O_2_^●−^, and/or able to impair tissue O_2_^●−^ and NO-derived peroxynitrite generation, could actually protect ALDH-2 from ROS inactivation. Vessels made tolerant by compounds **5** and **7** showed a weak rightward shift of the concentration–response curves (Figure 4b,d). For these molecules, devoid of the antioxidant function, was conceivable a behavior more similar to compounds **2** and **3** of the phenyl series. We hypothesized that the weak rightward shift observed in vessels made tolerant to compounds **5** and **7** was due to metabolic transformations. Indeed, it is known that in drugs containing the trimethoxyphenyl substructure, the *p*-methoxyl undergoes oxidative demethylation by the cytochrome P450 (CYP450). A similar metabolic transformation could also operate on derivatives **5** and **7**, regenerating the phenol with antioxidant properties. For this reason, we carried out an in vitro study of phase I metabolism, exploiting the microsomal fraction rich of CYP450, obtained from rat liver.

**Figure 12:**
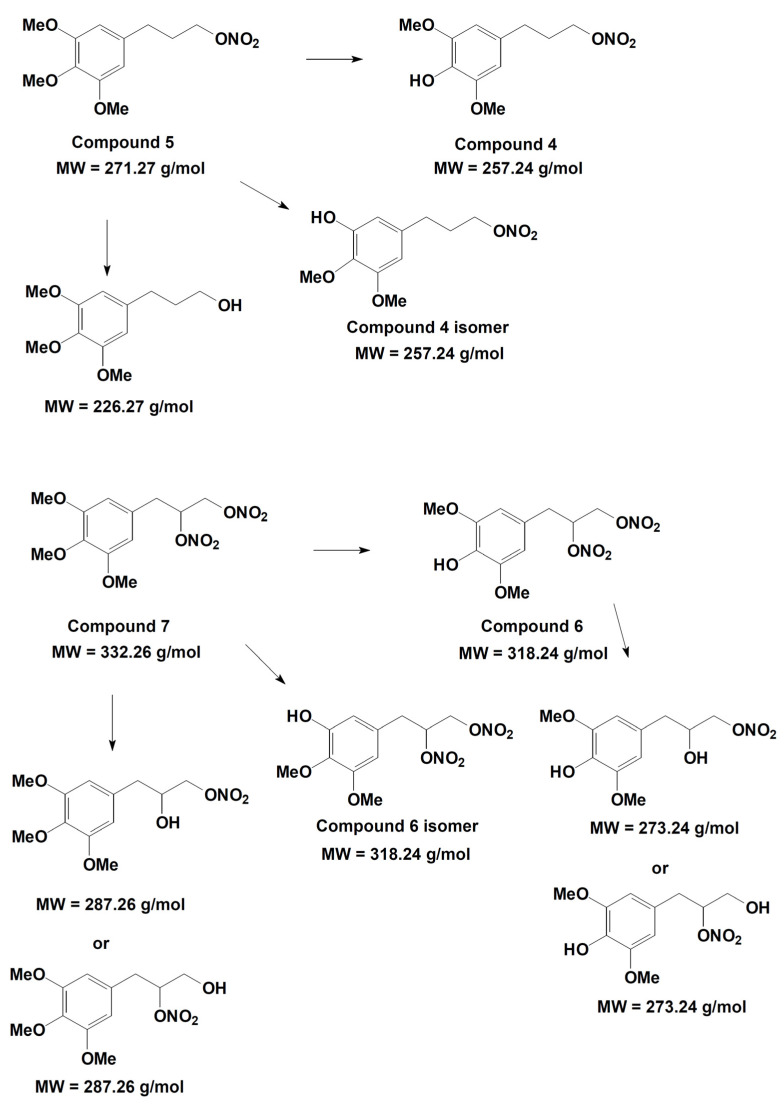
Metabolic pathway hypothesized for compounds **5** and **7**.

On the basis of the analytical data, the metabolic pathway reported in Figure 12 is proposed for compounds **5** and **7**. The identification of the metabolic products suggests that hydroxyl derivatives are produced by de-nitration of the side chain and also by de-methylation of both methoxy substituents present in the para- and meta- position with respect to the propyl-nitrate chain. The two isomers originating from the latter process can be easily distinguished on the basis of their well-separated retention time, even if they have the same molecular weight and similar mass spectrometric fragmentation. In the single de-nitration process involving compounds **6** and **7**, the analytical data cannot distinguish whether the process involved the nitro group located at the middle or at the extreme position of the side chain.

The in vitro study of phase I metabolism was aimed at verifying if the demethylated metabolites may partially protect the enzyme ALDH-2 by the action of ROS, decreasing the tolerance induced ex vivo by organic nitrates **5** and **7**. Likely, other metabolites may play a role in the vasodilating profile, but our aim was to explain why in ex vivo experiments compounds **5** and **7** did not induce a strong rightward shift of the concentration–response curves, as well as compounds **2** and **3**. The metabolism study confirmed that the weak rightward shift of the concentration–response curve observed in vessels made tolerant to compounds **5** and **7** might be due to the generation of metabolic derivatives demethylated in para position, that is **4** and **6**, endowed with antioxidant activity. 

## 5. Conclusions

In conclusion, the present study allows to deepen understanding of one of the complex mechanisms underlying the phenomenon of nitrate tolerance. Indeed, our results provide further evidence of the involvement of ALDH-2 in the development of tolerance. Moreover, the characterization ex vivo of the tolerance profile of organic nitrates with antioxidant properties allows us to support the hypothesis of the ROS involvement in inactivating ALDH-2. Indeed, antioxidant organic nitrates **4** and **6**, although bioactivated by ALDH-2, did not induce tolerance in our experimental model, unlike structurally similar derivatives **5**, **7** and **2**, **3**, which are devoid of antioxidant activity.

Our data provide fresh insight into the mechanisms responsible for nitrate tolerance, suggesting a potential role for multitarget drugs, namely antioxidant NO-donor organic nitrates, as a therapeutic tool in the prevention or control of the tolerance that accompanies the chronic use of GTN in patients.

## Figures and Tables

**Table 1:** Mass spectrometric acquisition parameters for the multiple reaction monitoring operating mode.

Compd	Precurson Ion (*m*/*z*)	Declustering Potential (V)	Entrance Potential (V)	Product Ions	Collision Energy (V)	Collision Cell Exit Potential (V)
**4**	258.2	30	4	258.2 → 212.2	12	15
				258.2 → 168.2	22	15
**5**	272.1	29	8	272.1 → 226.2	13	18
				272.1 → 182.2	20	14
				272.1 → 211.1	22	20
**6**	319.3	63	9	319.3 → 273.1	10	18
				319.3 → 167.0	18	16
				319.3 → 194.8	19	25
**7**	333.2	40	9	333.2 → 181.0	18	15
				333.2 → 167.1	34	30
				333.2 → 223.2	17	17

**Table 2:** Vasodilating activity of nitrooxyphenylalkyl derivatives and GTN.

	In Vitro Experiments pEC_50_ ± SE			Ex Vivo Experiments pEC_50_ ± SE	
Compd		+ Benomyl ^1^	+ Chloral Hydrate ^2^	Control	Tolerant Vessels
**1a**	6.68 ± 0.08 ^3^	6.49 ± 0.10 ^3^		6.92 ± 0.06	6.80 ± 0.05
**1b**	6.66 ± 0.07 ^3^	6.46 ± 0.10 ^3^	6.57 ± 0.03 ^3^	6.70 ± 0.12	6.49 ± 0.06
**2**	7.20 ± 0.15 ^3^	6.20 ± 0.12 ^3^	5.92 ± 0.3 ^3^	7.71 ± 0.11	6.67 ± 0.09 ^4^
**3**	6.80 ± 0.07 ^3^	5.82 ± 0.5 ^3^	5.52 ± 0.5 ^3^	7.36 ± 0.04	6.40 ± 0.06 ^5^
**4**	5.48 ± 0.09	4.85 ± 0.07	4.79 ± 0.11	5.48 ± 0.08	5.49 ± 0.05
**5**	5.52 ± 0.09	4.72 ± 0.07	4.68 ± 0.03	5.64 ± 0.08	5.22 ± 0.05 ^6^
**6**	6.19 ± 0.09	5.48 ± 0.08	5.59 ± 0.16	6.16 ± 0.04	6.06 ± 0.03
**7**	6.62 ± 0.10	5.58 ± 0.08	5.52 ± 0.06	6.55 ± 0.07	5.88 ± 0.02 ^7^
**GTN**	7.54 ± 0.04^3^	6.38 ± 0.07 ^3^	6.03 ± 0.05 ^3^	7.52 ± 0.04	6.10 ± 0.08

^1^ Experiments performed in the presence of 1 µM benomyl. ^2^ Experiments performed in the presence of 1 mM chloral hydrate. ^3^ Data previously published [23]. ^4^ *** *p* < 0.0001 vs. control (Student’s t test). ^5^ *** *p* < 0.0001 vs. control (Student’s t test). ^6^ *** *p* < 0.0003 vs. control (Student’s t test). ^7^ *** *p* < 0.0001 vs. control (Student’s t test). In vitro experiments: *n* = 4–7; ex vivo experiments: *n* = 9–21.

## Data Availability

Data is contained within the article and supplementary material.

## Supplementary Materials

The following are available online at https://www.mdpi.com/article/10.3390/antiox11010166/s1, Synthesis and characterization data for compounds **5** and **7**.
