# Supplementary material for: Multitarget Antioxidant NO-Donor Organic Nitrates: A Novel Approach to Overcome Nitrates Tolerance, an Ex Vivo Study
Source: Antioxidants (Basel). 2022 Jan 16;11(1):166. doi: 10.3390/antiox11010166 (PMC8773138; doi:10.3390/antiox11010166)
Supplement: Supplementary file 1 [file antioxidants-11-00166-s001.zip › antioxidants-1473955-supplementary.pdf]

Multitarget Antioxidant NO-Donor Organic Nitrates: A Novel Approach to Overcome Nitrates Tolerance, an Ex Vivo Study

Elisabetta Marini[1]*, Marta Giorgis[1], Marta Leporati[2], Barbara Rolando[1], Kostantin Chegaev[1], Loretta Lazzarato[1], Massimo Bertinaria[1], Marco Vincenti[3], Antonella Di Stilo[1]

[1] Dipartimento di Scienza e Tecnologia del Farmaco, Università degli Studi di Torino, via Pietro Giuria 9, I-10125 Torino, Italy

[2] Centro Regionale Antidoping e di Tossicologia "Alessandro Bertinaria", Regione Gonzole 10/1, I-10043 Orbassano, Italy

[3] Dipartimento di Chimica, Università degli Studi di Torino, via Pietro Giuria 5, I-10125 Torino, Italy

* corresponding author. Tel: +390116707196; e-mail: elisabetta.marini@unito.it

Supporting Materials

Chemistry. ^{1}H and ^{13}C NMR spectra were recorded on a BrukerAvance 300, at 300 and 75 MHz, respectively, using $SiMe_4$ as internal standard. The following abbreviations indicate peak multiplicity: *s* = singlet, *d* = doublet, *t* = triplet, *m* = multiplet. Low resolution mass spectra were recorded with a Finnigan-Mat TSQ-700. Flash column chromatography was performed on silica gel (Merck Kieselgel 60, 230−400 mesh ASTM). The progress of the reactions was followed by thin-layer chromatography (TLC) on 5 × 20 cm plates Merck Kieselgel 60 F254, with a layer thickness of 0.20 mm. Anhydrous sodium sulfate (Na_2SO_4) was used as drying agent for the organic phases. Organic solvents were removed under reduced pressure at 30 °C. Synthetic purity solvents dichloromethane (DCM), acetonitrile (CH_3CN), ethyl acetate (EtOAc), diethyl ether (Et_2O), 40−60 petroleum ether (PE) were used. Dry tetrahydrofuran (THF) was distilled immediately before use from Na and benzophenone under positive N_2 pressure. Commercial starting materials were purchased from Sigma-Aldrich, Alfa Aesar, and TCI Europe.

Synthesis of 3-(3,4,5-trimethoxyphenyl)propane-1,2-diyl dinitrate (**7**)

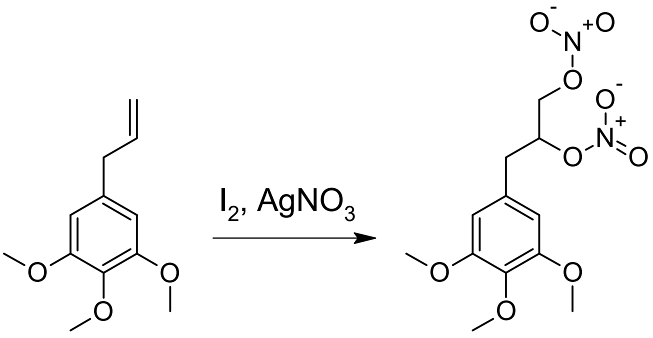

To a stirred solution of 5-allyl-1,2,3-trimethoxybenzene (1.80 g, 8.70 mmol) and AgNO3 (4.50 g, 26.0 mmol) in CH3CN (50 ml) iodine (2.20 g, 8.70 mmol) was added in one portion at rt. The reaction mixture was stirred at rt until all I2 dissolved and then under reflux for 48 h. After cooling, bine (10 mL) was added to the mixture and precipitate was filtered off and washed with EtOAc (100 mL). Filtrate was extracted with EtOAc (2 × 50 ml). The combined organic layers were washed with water, then brine, dried and evaporated. The resulting yellow oil was partly purified by flash chromatography (PE/EtOAc, 80/20 v/v). Obtained yellow oil was further purifiede by preparative HPLC (Lichrospher 250-25 C_{18}, CH_3CN/H_2O, 5/5 v/v mobile phase, flow-rate 25 mL/min, λ 254 nm) to give the title product as a yellow oil.

Yield: 24%. ^{1}H-NMR ($CDCl_3$) δ: 2.93 (*dd*, 1H, $^1J_{HH}$= 14.1 Hz, $^3J_{HH}$ = 7.2 Hz, ArC*H*H), 3.05 (*dd*, 1H $^1J_{HH}$= 14.1 Hz, $^3J_{HH}$= 6.9, ArCH*H*), 3.84 (*s*, 3H, CH_3O), 3.86 (*s*, 6H, 2CH_3O), 4.46 (*dd*, 1H, $^1J_{HH}$= 12.9 Hz, $^3J_{HH}$ = 6.3 Hz, C*H*HONO_2), 4.75 (*dd*, 1H $^1J_{HH}$= 12.9 Hz, $^3J_{HH}$= 3.0, CH*H*ONO_2), 5.41 – 5.49 (*m*, 1H, C*H*ONO_2), 6.43 (s, 2H, Ar); ^{13}C-NMR ($CDCl_3$) δ: 35.9, 56.2, 60.9, 70.2, 79.5, 106.1, 129.7, 137.5, 153.6. MS (EI) m/z 332 (M^+).

Synthesis of 3-(3,4,5-trimethoxyphenyl)propanyl nitrate (**5**)

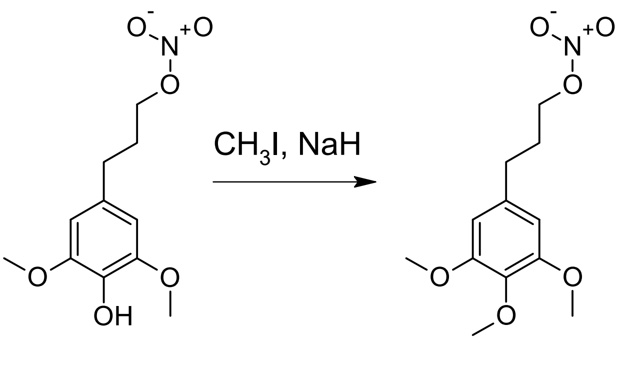

To a stirred solution of 3-(4-hydroxy-3,5-dimethoxyphenyl)propyl nitrate (330 mg, 1.28 mmol) in dry THF (10 mL) under positive N_2 pressure NaH 60% in mineral oil suspension (50 mg, 1.28 mmol)

was added, followed after 10 min by CH_3I (480 µL, 7.68 mmol). The reaction mixture was stirred at rt until completed (TLC control). Than it was diluted with Et_2O (20 mL) and organic layer was washed with water, then brine, dried and evaporated. The resulting yellow oil was purified by flash chromatography (PE/EtOAc, 90/10 v/v). to give the title product as a yellow oil. Yield: 55%.
^{1}H-NMR ($CDCl_3$) δ: 2.06 (*m*, 2H, $CH_2CH_2CH_2$), 2.70 (*t*, 2H, $^3J_{HH}$= 7.7, ArCH_2), 3.84 (*s*, 3H, CH_3O), 3.86 (*s*, 6H, 2CH_3O), 4.47 (*t*, 2H, $^3J_{HH}$ = 6.3 Hz, CH_2ONO_2), 6.40 (s, 2H, Ar); ^{13}C-NMR ($CDCl_3$) δ: 25.9, 29.2, 56.2, 60.9, 71.3, 106.1, 129.
